# Assessing Biases in the Evaluation of Classification Assays for HIV Infection Recency

**DOI:** 10.1371/journal.pone.0139735

**Published:** 2015-10-05

**Authors:** Oscar Patterson-Lomba, Julia W. Wu, Marcello Pagano

**Affiliations:** 1 Department of Biostatistics, Harvard T.H. Chan School of Public Health, Boston, United States of America; 2 Department of Epidemiology, Harvard T.H. Chan School of Public Health, Boston, United States of America; University Medicine Greifswald, GERMANY

## Abstract

Identifying recent HIV infection cases has important public health and clinical implications. It is essential for estimating incidence rates to monitor epidemic trends and evaluate the effectiveness of interventions. Detecting recent cases is also important for HIV prevention given the crucial role that recently infected individuals play in disease transmission, and because early treatment onset can improve the clinical outlook of patients while reducing transmission risk. Critical to this enterprise is the development and proper assessment of accurate classification assays that, based on cross-sectional samples of viral sequences, help determine infection recency status. In this work we assess some of the biases present in the evaluation of HIV recency classification algorithms that rely on measures of within-host viral diversity. Particularly, we examine how the time since infection (TSI) distribution of the infected subjects from which viral samples are drawn affect performance metrics (e.g., area under the ROC curve, sensitivity, specificity, accuracy and precision), potentially leading to misguided conclusions about the efficacy of classification assays. By comparing the performance of a given HIV recency assay using six different TSI distributions (four simulated TSI distributions representing different epidemic scenarios, and two empirical TSI distributions), we show that conclusions about the overall efficacy of the assay depend critically on properties of the TSI distribution. Moreover, we demonstrate that an assay with high *overall* classification accuracy, mainly due to properly sorting members of the well-represented groups in the validation dataset, can still perform notoriously poorly when sorting members of the less represented groups. This is an inherent issue of classification and diagnostics procedures that is often underappreciated. Thus, this work underscores the importance of acknowledging and properly addressing evaluation biases when proposing new HIV recency assays.

## Introduction

Properly identifying recent HIV infection cases is important from a public health and clinical perspectives [1]. It is essential for accurately estimating incidence rates, which are in turn critical for monitoring transmission dynamics, identifying groups at high risk of infection, and determining the impact of interventions [2–5]. Detecting individuals in the early stages of infection is also crucial for HIV prevention as recently infected individuals are linked to a disproportionate share of transmission events. Furthermore, early onset of antiretroviral treatment has shown to improve disease progression outlook and reduce the risk of further transmission [6–8].

However, the direct measurement of individuals’ infection stage and incidence rates is expensive and time-consuming given the need to repeat serological testing of a cohort of individuals over time. In addition, this cohort may be subject to methodological and observational challenges which can limit its representativeness of the overall population [9].

A supplemental and promising means to identify recently infected individuals comes through the development of methods that, focusing on time-dependent biomarkers such as viral sequence diversity, can distinguish recent from chronic infections on the basis of cross-sectional serological specimens [4, 10–12]. However, these methods have other issues, including great variation in biomarker evolution across people due to differential immune response, antiretroviral therapy use and multiplicity of infection (infection by more than one founder viral strain). In addition, and more relevant to this work, these methods can also be limited by the characteristics of the subjects in the sample used to evaluate them.

The issue of how properties of the validation sample can elevate or decrease the efficacy of a given diagnostic test was addressed decades ago in [13], where it is noted that to establish a test efficacy, certain features of the patients examined in the study should be representative of those in the population of interest. In this work, where we address the issue of using biomarkers to classify HIV infected patients as recently infected or not, an important feature of the patients is the *time since infection* (TSI).

**Fig 1:**
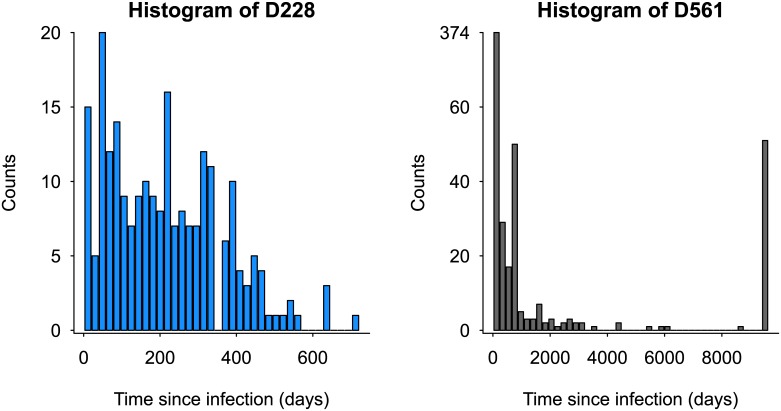
Empirical *time since infection* distributions of two available datasets. On the left, D228 represents 228 samples (from 42 subjects) with recent infection of subtype C in Botswana from 2004 to 2008. Subjects were followed longitudinally for no more than 755 days [17]. On the right, D561 represents a meta database (freely available at Los Alamos HIV public database; accessed August 2014) of 561 samples (from 462 subjects) with subtype B and C. The maximum TSI is 8888 days.

Two datasets, referred herein as D228 and D561, containing viral genetic sequences from HIV infected patients as well as their estimated time of infection, have been used in the design and validation of classification algorithms to determine recency of infection based on within-host viral genetic diversity [11, 14–16]. D228 and D561 contain 228 and 561 person-time points of *gag* and *env* genes viral sequence data, respectively. The respective TSI distributions are depicted in Fig 1, where it is easy to detect important differences between these two distributions. For instance, their respective shape and ranges are quite different (with D228 having a narrower range as well as being more concentrated to the more recent cases). Can these differences significantly affect the performance of HIV recency classification algorithms that use this datasets for validation purposes? If we compare two classification algorithms that have used considerably different datasets to evaluate their performance, is this a reasonable comparison? How does the TSI distribution of the recruited patients affect the performance of an HIV recency classification algorithm? These are the types of questions that will be addressed in this study.

Developing an assay requires the discovery of new biomarkers (e.g., a measure of viral diversity) and the design of a classification algorithm that combines these biomarkers as to render a model with high predictive power. These procedures need a training dataset to study the proposed models and a validation dataset to assess how the predictive power of each model generalizes to an independent dataset. In this paper, we assume that a useful biomarker and proper model have already been developed. What we then assess is how the evaluation of this hypothetical biomarker/model “package” is affected by the nature of the validation dataset. In this paper we refer to an *assay* as the combination of both the biomarker and the classification algorithm.

## Materials and Methods

### TSI distributions

We compare the performance of a classification assay using six TSI distributions: four simulated TSI distributions representing different epidemic scenarios, and the TSI distributions of the two empirical datasets described above in Fig 1. To that end, we first define the simulated distributions.

Let the time since infection (TSI) be a random variable *T*, measured in days, with *t*_*max*_ = max(*T*) and *t*_*min*_ = min(*T*). Let also *T* = *g*(*X*) = Δ*TX* + *t*_*min*_, with Δ*T* = *t*_*max*_ − *t*_*min*_. Here *X* is a Beta distributed random variable with probability density function given by *F*_*X*_(*x*, *a*, *b*) with parameters *a* and *b*. Since the function *g* is monotonic, then the density function of *T*, with random realization *t*, is given by

$$F_{T}\;\left(t,a,b\right)=|\frac{d}{dt}\left(g^{-1}\;\left(t\right)\right)|\cdot F_{X}\;\left(g^{-1}\;\left(t\right)\right)=\frac{1}{\Delta T}F_{X}\;(\frac{t-t_{min}}{\Delta T},a,b),$$ (1)

where *g* ^−1^ denotes the inverse function.

**Fig 2:**
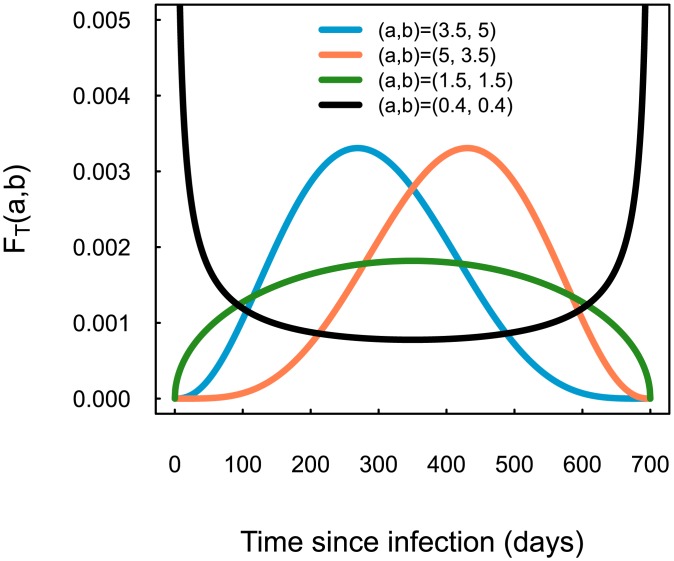
Hypothetical Time Since Infection distributions. These distributions have a Beta distribution kernel with parameters *a* and *b*. They are meant to represent different epidemic scenarios (akin to those in [18]). The blue line represents the case of an “emerging” epidemic; the orange line a “waning” epidemic; the green line a “stable” epidemic; and the black line an epidemic that has been partially controlled for a period of time, but has recently resurged. The latter scenario is arguably the least likely to be found in reality, and we have included it to mimic the properties of the TSI distribution of D561 in Fig 1.

Using the Beta distribution as a kernel for the TSI distribution allows for the shape of this distribution to be considered as a descriptor for different sampling populations of HIV infected patients. In turn, the structure of these sampling distributions may be related to different epidemic scenarios [18] (e.g., emerging or waning epidemics) present in the population. Fig 2 shows some of these scenarios.

Noteworthy, the shape of the TSI distribution may be also subject to sampling and/or observational biases due to selective recruitment, follow-up attrition, as well as data manipulation. The latter can occur in cases where, due to the difficulty of collecting the data (as in the case of viral sequence data), each study typically generates a relatively small dataset. Consequently, the data used to evaluate a recency assay is scant, and aiming to increase sample size and statistical power, investigators are keen to combine data from several studies to construct a “convenience sample” to evaluate their assay. However, this pooled dataset can yield datasets with unrealistic features, potentially affecting algorithm performance. In fact, dataset D561 is a case of a “convenience sample”.

### Model for evolution of biomarker: within-host viral diversity

We know that some measures of within-host HIV viral diversity increase fairly consistently during the first few months of infection, later reaching a plateau, and even decreasing in more advanced infection stages [19, 20]. These patterns, however, show high degree of variability among hosts [11, 18, 20, 21]. Some of the reasons for this variability, besides the differences in the innate immune response of each individual, are treatment status, multiplicity of infection and strain type [14].

**Fig 3:**
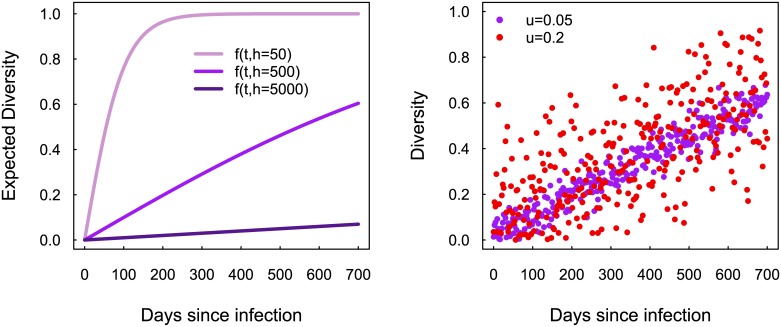
(**left**) Expected diversity evolution for different model behaviors, as determined by parameter *h* in Eq (3). (**right**) Diversity values of model *f*(*t*) with *h* = 500 for two standard deviation values: *u* = 0.05 (purple) and *u* = 0.2 (red). The profiles of real data commonly relate more to the case of *u* = 0.2.

For this study, we assume that, on average, the viral diversity of all infected subjects follow the same temporal pattern with some variability added in the form of an error term to account for measurement errors and biological stochasticity. Without loss of generality, we assume our diversity metric to be between 0 and 1, such as in the case of diversity measured via Shannon entropy [11, 22]. More specifically, we propose that the diversity measure *d*_*i*_ ∈ (0,1) of patient *i* whose TSI is given by *t*_*i*_, is

$$d\left(t_{i}\right)=d_{i}=f\left(t_{i}\right)+\epsilon_{i},$$ (2)

where *ϵ*_*i*_ is a normally distributed error term with a distribution *N*(0, *u*), truncated such that 0 < *f*(*t*_*i*_) + *ϵ*_*i*_ < 1 for all *i*. Therefore, the expected diversity is $E[d_{i}]=f(t_{i})$. Based on empirical considerations, the functional form of *f*(*t*) is selected such that 0 < *f* < 1, and *f* increases monotonously for *t* ∈ (0,∞) plateauing for large values of *t*. Although a number of functions are possible, here we propose:

$$\begin{array}{ccc} f(t) & = & \frac{2}{1+\exp(-t/h)}-1, \end{array}$$ (3)

akin to that proposed in [5]. As we can see in Fig 3 (left), the *h* parameter controls the rate of increase with respect to time. Fig 3 (right) shows how increasing the standard deviation, *u*, of the error term in Eq (2) increases the variability in the simulated data.

The form of *d*(*t*), that is, the temporal evolution of the selected biomarker, depends greatly on how well the biomarker is defined or constructed. For example, a “better” biomarker would be one with less variability and greater average temporal increase. Indeed, a smaller value of the variability term *u* would improve the predictive ability of the biomarker (see Tables A and B in S1 Text). Given that in reality there will always be some level of inherent stochasticity and measurement error such that *u* > 0, an intermediate value of *h* would guarantee that *f*(*t*) does not grow too slowly or plateau too quickly, thus increasing the differentiability between recent and chronic cases. That said, we note that in this work we are not assessing the merits of a specific biomarker, but rather we are investigating the effects that the nature of the TSI distribution has on the performance of a hypothetical biomarker whose temporal behavior is described by *d*(*t*) with *h* = 500 and *u* = 0.2.

### Classification algorithm

For the classification algorithm we use a univariate logistic regression [23] where the status of individuals (recent = 1 or chronic = 0) are regressed on their respective diversity measures. We then report performance metrics such as the area under the receiver-operating characteristic (ROC) curve (i.e., the AUC) as a single quantitative index of the assay’s classification accuracy [24]. Since we are interested in assessing the performance of the assay at the individual and the population levels, we also report sensitivity and specificity (as individual-level performance metrics), as well as accuracy and precision (as population-level performance metrics, more relevant for incidence estimation) [5]. Sensitivity (specificity) is defined as the proportion of recent (chronic) cases classified as such by the algorithm. In this study we estimate sensitivity and specificity as to be simultaneously maximized (formally equivalent to maximizing of the Youden’s J statistic). Accuracy is the proportion of true results in the whole sample. Precision (or positive predictive value), an arguably more useful metric for clinicians, is defined as the proportion of positive tests that are identified as recent cases, and it is not only a function of the efficacy of the assay, but also of the prevalence of recent cases [25].

### Assessing algorithms based on different TSI categories

In addition to quantifying the *overall* performance of the algorithms, we can report classification performance metrics for different types of individuals (i.e., individuals that belong to different TSI categories or strata). In other words, we can report performance metrics as a function of TSI. In this way, we can present a more useful and complete assessment of the assay’s performance [4, 21, 26].

Given a TSI distribution *F*(*t*), a model for within-host diversity evolution *d*(*t*), and a recency cutoff time, t*, we can simulate pairs of the form (*s*_*i*_, *d*_*i*_), where the status *s*_*i*_ is recent (*s*_*i*_ = 1) if *t*_*i*_ ≤ t* or chronic (*s*_*i*_ = 0) if *t*_*i*_ > t*, and *d*_*i*_ = *d*(*t*_*i*_). These pairs are regressed using logistic regression, yielding a model fit from which the ROC curve can be drawn. From this ROC curve we can determine performance metrics such as AUC, sensitivity and specificity.

However, the values of sensitivity and specificity, depend on a diversity cutoff value, d*, that is, the biomarker cutoff or *classifier*. If *d*_*i*_ < d*, the algorithm classifies the patient as recent, or chronic otherwise. Assuming our objective function gives equal weights to sensitivity and specificity, the d* cutoff is obtained from the expression

$$\max\left[\text{sensitivity}\left(d\right)+\text{specificity}\left(d\right)\right]=\text{sensitivity}\left(d^{*}\right)+\text{specificity}\left(d^{*}\right).$$ (4)

Once we have obtained d* we can determine a function that quantifies the probability of providing a correct classification as a function of the TSI. That is, letting $s_{i}^{e}$ and $s_{i}^{a}$ be the *estimated* (or predicted) and *actual* recency status of subject *i* with TSI given by *t*_*i*_, we then want to estimate the probability

$$p\left(\tau \right)=P\left[s_{i}^{e}=s_{i}^{a}|t_{i}=\tau \right].$$ (5)

Therefore, sensitivity, which is commonly defined as the *overall* probability of identifying a case as recent given that the case is recent, is related to *p*(*τ*) by

$$\text{sensitivity}=\frac{\int_{0}^{t^{*}}p\left(\tau \right)F\left(\tau \right)d\tau }{\int_{0}^{t^{*}}F\left(\tau \right)d\tau }.$$ (6)

By the same logic, specificity is related to *p*(*τ*) by

$$\text{specificity}=\frac{\int_{t^{*}}^{\infty }p\left(\tau \right)F\left(\tau \right)d\tau }{\int_{t^{*}}^{\infty }F\left(\tau \right)d\tau }.$$ (7)

In practice, however, given the paucity of the data, it is difficult to estimate the function *p*(*τ*) for a particular value of *τ*. We can instead estimate the function for TSIs that fall within a given range (*τ*_1_, *τ*_2_), where *τ*_1_ < *τ*_2_ < t* or t* < *τ*_1_ < *τ*_2_. That is, we can estimate

$$p\left(\tau_{1},\tau_{2}\right)=P\left[s_{i}^{e}=s_{i}^{a}|t_{i}\in \left(\tau_{1},\tau_{2}\right)\right].$$

In words, *p*(*τ*_1_, *τ*_2_) is the probability of correctly classifying a case whose TSI is between *τ*_1_ and *τ*_2_.

The way we implement this estimation is as follows. We define a binning structure (or stratification) for subjects based on their TSI, as to have *K* different categories (or bins) of subjects [4]. Subjects with TSI given by *t*_*i*_ ∈ (*τ*_*k*_, *τ*_*k*+1_), with *k* = {1, 2…*K*}, belongs to the *k* ^*th*^ category. For subjects in the *k* ^*th*^ category, with *τ*_*k*_ < *τ*_*k*+1_ < t*, we estimate the sensitivity as a function of TSI as the proportion of these subjects whose diversity measure is lower than the diversity cutoff d* (as defined in Eq (4)). More formally,

$${\hat{p}}_{k}=\frac{\text{subjects}\;\text{in}\;\text{the}\;k^{th}\;\text{category}\;\text{with}\;d_{i}<d^{*}}{\text{all}\;\text{subjects}\;\text{in}\;\text{the}\;k^{th}\;\text{category}}.$$ (8)

Similarly, for subjects in the *k* ^*th*^ category, with t* < *τ*_*k*_ < *τ*_*k*+1_, we estimate the specificity as a function of TSI as the proportion of these subjects whose diversity measure is higher than d*. Note that with this discretization of the *p*(*τ*) function, the overall sensitivity and specificity given in Eqs (6) and (7) are approximated, respectively, by

$$\text{sensitivity}=\frac{\sum_{k\in K_{1}}{\hat{p}}_{k}T_{k}}{\sum_{k\in K_{1}}T_{k}}\;\text{specificity}=\frac{\sum_{k\in K_{2}}{\hat{p}}_{k}T_{k}}{\sum_{k\in K_{2}}T_{k}}$$ (9)

where *T*_*k*_ is the fraction of individuals in category *k*, and *K*_1_ and *K*_2_ are the sets of categories where *τ*_*k*+1_ < t* and *τ*_*k*_ > t*, respectively, with *K* = *K*_1_ ∪ *K*_2_. We can then ask: how does ${\hat{p}}_{k}$ depend on the TSI distribution?

Noteworthy, since the probability that an observation with *τ*_*k*_ < t* (> t*) is classified as “recent” (“chronic”) is 1 by definition, the amount of error or bias in accuracy in each bin can be quantified by $1-{\hat{p}}_{k}$.

Estimating the curve *p*(*τ*) in Eq (5) is important for incidence estimation given its relation to the probability of classifying as “recent” an individual with TSI equal to *τ*, namely *r*(*τ*). In fact *r*(*τ*) = *p*(*τ*) for *τ* < t* and *r*(*τ*) = 1 − *p*(*τ*) for *τ* ≥ t*. The curve *r*(*τ*) is used for the estimation of incidence from cross-sectional data via quantities such as the *mean duration of recent infection* and the *false-recent rate*[5, 21, 26]. The first quantity is given by $\int_{0}^{t^{*}}r\left(\tau \right)d\tau$, whereas the second one is equivalent to 1-specificity, which is determined by *p*(*τ*), see Eq (7). Likewise, the *mean window period* and the *shadow*, quantities that are useful for computing incidence using estimates of recent cases, are also tied in to *r*(*τ*) [4, 10]. Given the close relationship between *p*(*τ*) and *r*(*τ*), we can conclude that if the curve *p*(*τ*) is affected by the shape of TSI distribution of the validation sample, then all these other quantities will also be affected.

#### Simulation steps

The results shown below are obtained following a series of steps. When simulations use the simulated TSI distributions described above (Fig 2), the procedure is:

1. Select the TSI distribution
2. Sample *N* times from this distribution, obtaining *N* TSI values *t*_1_, *t*_2_,…, *t*_*N*_
3. Get the status of each of the *N* individuals by converting these TSI values into ones if *t*_*i*_ < t* or zeros otherwise
4. Convert these TSI values into the corresponding *N* diversity values using *d*(*t*)
5. Regress *status* ∼ *diversity* using univariate logistic regression
6. Compute performance metrics: AUC, sensitivity, specificity, accuracy and precision

When simulations use the empirical TSI distributions shown in Fig 1, we proceed similarly. The only change is in step 2, where *N* is equal to the sample size of the particular dataset (228 or 561 samples) and there is no need for sampling.

To obtain mean performance values and their corresponding prediction intervals, after having selected the distribution and the sample size *N*, we repeat steps 2–6 *S* times. From these *S* values of each performance metric we compute their means and its 95% prediction intervals (i.e., the range of values that contain 95% of the observations, which reflects the variation of the data and is formally defined by *μ* ± 1.96*σ*, with *μ* and *σ* being the mean and standard deviation of the observations). For the simulated distributions we set *N* = 500. To obtain mean performance values and their respective prediction intervals we repeat the procedure *S* = 1000 times. We investigated the effect of the sample size on our analyses and concluded that for *N* ≥ 500 neither the mean behavior nor the prediction intervals change considerably. We also found that increasing the number of simulations from *S* = 1000 to *S* = 5000 did not decrease the size of the prediction intervals in a significant way.

Finally, we note that since we can perform any large number of simulations, the 95% *confidence* intervals around the mean (defined by $\mu \pm 1.96\frac{\sigma }{\sqrt{S}}$) tend to zero as we increase *S*. With *S* = 1000 simulations we obtained 95% confidence intervals around the mean of all metrics that are negligible in size. Therefore, we can conclude that with 1000 simulations we effectively know the population mean of the performances, hence any differences that we observe in the mean of the performances are statistically significant.

All statistical analyses presented in this work were performed in the **R** statistical software package [27].

## Results

We study how the TSI distributions of the patients affect the performance of an HIV recency assay. Motivated by the differences in shape and range seen in the empirical TSI distributions in Fig 1, we explore different parametrizations (shapes and ranges) of the simulated TSI distributions (Fig 2), in addition to the two empirical distributions.

### The impact of TSI distribution on assay performance

**Fig 4:**
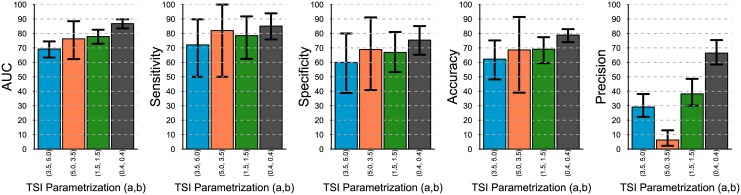
Comparing classification performance of the same assay with different TSI distributions. With *u* = 0.2, sample size = 500, *h* = 500, recency at 6 months. The 95% prediction bounds are obtained from 1000 simulations.

By comparing the results in Fig 4 we can readily conclude that when the TSI distribution has a bimodal shape (dark gray bars), the assay features, on average, the best classification performance in terms of all the metrics presented. This is mainly due to the better identifiability of recent from chronic cases in such distribution. At the opposite end, when the TSI distribution is right-skewed (blue bars), the assay renders its worst average performance (in terms of all the metrics except for precision), presumably due to the lack of differentiability between recent and chronic cases. The low precision values obtained when using the left-skewed distribution (orange bar) is due to the low prevalence of recent cases in such distribution.

Finally, the prediction intervals around the means indicate that the variability of the assay is lower when the TSI distribution is bimodal, and it is larger when the TSI has a left-skewed distribution.

### The impact of TSI distribution *and* definition of recency on assay performance

Different authors use different definitions of recency. Two of the most common definitions are delimiting recency at 6 months or at 1 year. Here we investigate the impact of recency definition, in combination with different TSI distributions, on classification performance.

**Fig 5:**
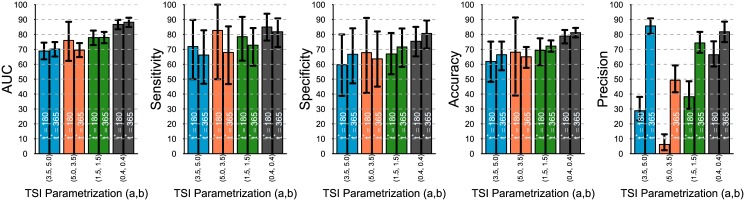
Comparing classification performance of the same assay with different TSI distribution and two different definitions of recency: at t* = 180 days (left bars) and t* = 365 days (right bars). With *u* = 0.2, sample size = 500, *h* = 500. The 95% prediction bounds are obtained from 1000 simulations.

From Fig 5 we can conclude that the average classification performance, as expressed by the AUC, specificity and accuracy, improves slightly when recency is defined at 1 year versus 6 months, except for the case where the TSI distribution has less recent cases (orange bars). The sensitivity, on the contrary, decreases when recency is defined at 1 year.

In terms of the precision, the improvements are systematic and substantial, and are mainly due to the increase in the prevalence of recent cases under this definition of recency.

### The impact of TSI distribution *and* TSI range on assay performance

One of the key differences between dataset D561 and D228 is that their ranges are starkly different: 4 to 755 days for D228 versus 14 to 8888 days for D561. Thus, in this section we investigate the effect of varying the range Δ*T* of the TSI distributions (or more precisely, varying *t*_*max*_ from 700 to 1400 days while keeping *t*_*min*_ = 1 day), while maintaining the same kernel parametrization (that is, same combinations of *a* and *b* as before).

**Fig 6:**
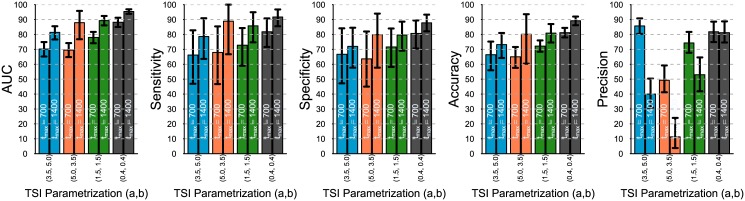
Comparing classification performance of the same assay with different TSI distribution and different ranges of TSI: 1 day up to *t*_*max*_ = 700 days (left bars) and up to *t*_*max*_ = 1400 days (right bars). With *u* = 0.2, sample size = 500, *h* = 500, recency at 365 days. The 95% prediction bounds are obtained from 1000 simulations.

Fig 6 readily shows that when the assay is evaluated on the data with a larger TSI range, on average, the assay’s classification performance is significantly improved, except for its precision, which is consistently affected presumably due to a smaller fraction of recent cases in a more “stretched” distribution.

### Assessment on TSI distributions from empirical datasets

In this section we investigate the impact of the TSI distribution on performance using the two empirical distributions from datasets D228 and D561 (Fig 1). As previously specified in the Simulation Steps, in this case the diversity measures come from the same model of within-host diversity evolution, *d*(*t*), the only difference being that *d*(*t*) operates on the TSI from the empirical datasets.

**Fig 7:**
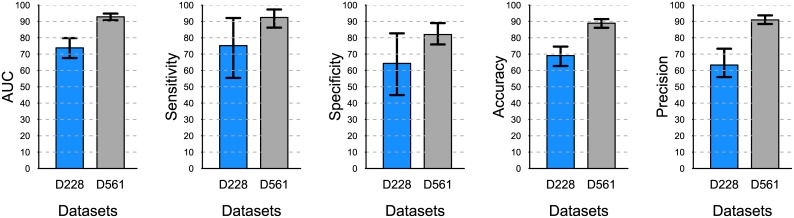
Classification performance of the same assay using the two TSI distributions from the empirical datasets. With *u* = 0.2, *h* = 500, recency at 6 months. The 95% prediction bounds are obtained from 1000 simulations.

Fig 7 shows that the performance of the recency assay differs significantly depending on the TSI distribution of the dataset used. Corroborating the previous findings, the assay features greater performance and lower variability on the D561 dataset, whose TSI distribution has a bimodal shape and larger range when compared to that of D228.

### Assessing the assay’s performance at different TSI categories

The ultimate goal of a recency assay is not to correctly classify patients within the validation set, but to provide an effective metric to help investigators classify patients in an specific target population. By providing a performance measure for each type of individual (based on his/her TSI category) we can offer a more refined assessment of the algorithm performance.

To do so we apply Eq (9) and use the TSI distributions from the empirical datasets D228 and D561. The question here is: how does the assay performance for each TSI category depend on the TSI distribution of the validation datasets (in this case the D228 and D561 distributions)?

To compare the assay’s performance on the these two datasets, we stratified the samples based on their TSI into the following categories: 1–2 months, 3–4 months…,19–20 months, and 21+ months. Noteworthy, the last category is much more populated for D561 (141 cases) than for D228 (4 cases).

**Fig 8:**
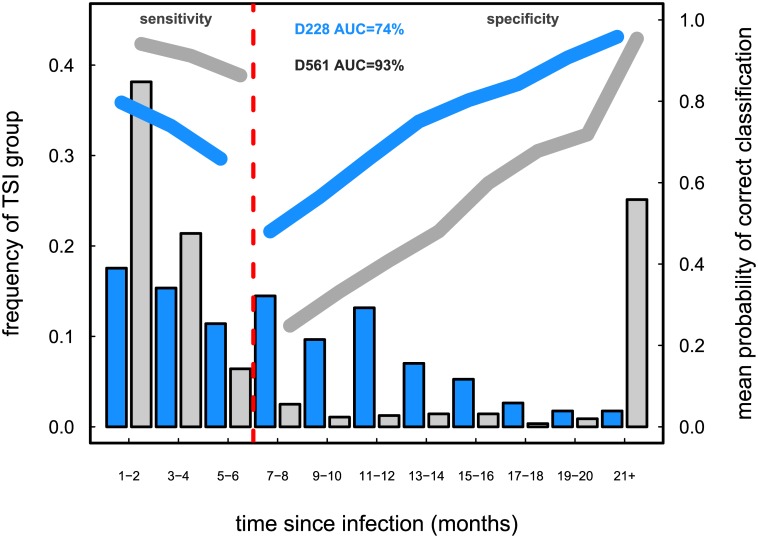
Comparing the mean performance (i.e., mean of ${\hat{p}}_{k}$ over *S* = 1000 simulations) of the same hypothetical HIV recency assay using the two empirical TSI distributions (note different *y* axis, with the left *y* axis representing the frequency of each of the 2-month TSI groups or bins, and the right *y* axis representing the mean of ${\hat{p}}_{k}$). The assay performs better overall in the dataset D561 which has a bimodal TSI distribution (dark gray, AUC = 93%) than in dataset D228, which has a TSI distribution with a large fraction of cases around the recency cutoff of 6 months (blue, AUC = 74%). In addition, the assay’s sensitivity as a function of TSI is also higher in dataset D561, however, its specificity is higher in dataset D228. For the case of D561, in the range of 6 to 15 months the assay does worse, on average, than a “coin toss” (mean probability < 0.5). Parameter values: *u* = 0.2, *h* = 500.

The mean assay performance when using the TSI distributions from D228 (blue) and D561 (dark gray) is presented in Fig 8. The picture shows that the assay evaluated with the D561 dataset features a higher AUC (overall performance) and higher sensitivity, but it also has a lower specificity, compared to the same assay evaluated with D228. A qualitatively similar behavior is obtained when using the simulated TSI distributions instead of the empirical ones (see Figure A in S1 Text).

To better understand why the assay validated with D561 features a comparatively poor accuracy for individuals whose TSI are larger than 6 months, even though its *overall* performance is much higher than when validated with D228, we must turn our attention to Eq (9). The overall performance is a combination of the estimated ${\hat{p}}_{k}$ and the corresponding *T*_*k*_. In other words, what the classifier “tries” to maximize is ∑_*k*_ *p*_*k*_ *T*_*k*_, not ∑_*k*_ *p*_*k*_. For instance, when using D561, ${\hat{p}}_{k}$ is larger wherever *T*_*k*_ is also large (where a large fraction of the cases lie), whereas ${\hat{p}}_{k}$ is lower when *T*_*k*_ is also small. The result is that the assay validated with D561 performs better overall.

It is import to remark how in Fig 8 the bias in the ${\hat{p}}_{k}$ estimates (with the bias given by $1-{\hat{p}}_{k}$) tends to be lower for highly represented TSI groups or bins, and vice versa, the reason being that as we aim to maximize the overall accuracy of the assay, bins that contain a large fraction of the observations are more likely to get a less biased estimate of *p*_*k*_. Moreover, Figure G in S1 Text illustrates how the variability around ${\hat{p}}_{k}$ is also lower for highly populated bins, which is due to the variability in a given bin largely depending on the number of observations in that bin.

The binning structure can also affect the estimation of *p*_*k*_. However, if the estimates of *p*_*k*_ do not vary abruptly from month to month, then the binning structure becomes less of an issue. In fact, we binned the empirical data in 3-month bins, instead of 2-month bins as used in Fig 8, and found no important differences in the estimates of *p*_*k*_ when comparing the results from these two binning criteria.

## Discussion

Proper identification of recent HIV infections is essential for estimating HIV incidence rates, as well as for reducing transmission risk and improving clinical outlook of patients. Crucial to this task is the development of robust and generalizable classification assays that, based on cross-sectional viral sequence data, help determine infection *recency* status.

To guarantee a proper (unbiased) evaluation and applicability of a given assay it is critical to ensure that the validation dataset is representative of the demographic and epidemiological context in which the assay will be employed [4, 5, 26]. This task is particularly challenging given the scarcity of viral sequence data, which lead researches to aggregate data from several studies to construct “convenience samples” in order to increase sample size and statistical power. This practice, however, can render datasets with structures that are not representative of the target population.

In this work we show that the performance of an HIV recency classification assay– when measured using both population-level and individual-level performance metrics– may be critically determined by the nature of the validating data at hand. An important aspect of this data is the time since infection (TSI) distribution of the infected individuals.

Using four simulated TSI distributions that represent different epidemiological scenarios, our analyses show that when the TSI distribution of the validation sample has a bimodal shape and larger TSI range, the assay performs at its best (i.e., higher mean performance and lower variability) given the higher differentiability of recent from chronic cases. Conversely, in terms of its AUC, the assay performs at its worst when the TSI distribution is right-skewed, particularly when recency is defined at 6 months, given that most cases are concentrated around the recency cutoff, thus diminishing identifiability. This result suggests that an assay that uses the D228 dataset (also right-skewed) to evaluate its performance, might not render high overall classification power. Meanwhile, in terms of its precision, the algorithm shows its worst performance when the TSI distribution is left-skewed and has a longer range, given the low prevalence of recent cases.

The results using the two empirical datasets corroborate that datasets with bimodal and wider TSI distributions render better assay performance and stability. The fact that D228 TSI distribution has a less differentiable shape and smaller range, as compared to D561, are some of the reasons why assays are shown to perform better when using dataset D561 [16] when compared to those using D228 [11]. These results underscore the importance of specifying the TSI distribution of the validation dataset when introducing new assays.

Finally, evaluation of the assay’s performance at different TSI categories in the empirical datasets shows that, even though the assay performs better in terms of its AUC and sensitivity when evaluated with the bimodal dataset (D561), its specificity is lower than that of the assay evaluated with the right-skewed distribution (D228). This finding should not come as a surprise. The curves depicting the probability of correct classification for both datasets demonstrate two general aspects in classification problems that are relevant in practice: classification performance decreases near the TSI threshold that divides people into recent or chronic, and the assay’s classifier performs better (i.e., less bias and variability) for the types of individuals whose TSIs are more represented in the validation sample.

The first aspect is inherent to the problem of classification, and it will always be present to some extent. It is also a key reason why the overall performance is relatively low when the individuals in the validation sample are distributed such that a large fraction of the population is near the threshold (as in the case of D228).

The second aspect is a result of the classification algorithm incorporating biases present in the validation dataset, with one important consequence being a lower accuracy for the type of individuals that are less represented in such dataset. The flip side of this aspect is that, in general, these classification algorithms tend to feature higher rates of accuracy for those individuals who belong to the statistically dominant groups (in the case of D561, individuals with TSI between 1 to 4 months, or much larger than 21 months). As such, when the algorithm is evaluated using D561, its overall prediction accuracy is high, even though its performance is notoriously poor for individuals whose TSI lies within 6 to 15 months. Correctly classifying these types of individuals as chronic cases should be a key priority of any HIV recency classification assay.

These findings imply that the estimation of the probability of correct classification, *p*(*τ*), is influenced by the shape of the TSI distribution, as shown in Fig 8 and Figure F in S1 Text. A key reason being that the TSI distribution plays a key role in determining the biomarker cutoff, d*, which dictates whether an observation is classified as recent or not (see Figure B in S1 Text). Consequently, other metrics such as the mean duration of recent infection, false-recent rate, mean window period and the shadow, all essential for the proper estimation of incidence from cross-sectional data, can also be affected by the shape of the TSI distribution.

These issues become particularly worrisome if the TSI distribution of the validation sample is *not* representative of the target population (such as those in some “convenience samples”), but yet it is “nicely behaved” as to render a high *overall* classification performance on this non-representative sample. This lack of representativeness can result in an assay that misclassifies the type of individual that constitutes a considerable fraction of the *target* population, thus performing rather poorly in practice. Noteworthy, it is often difficult to have prior knowledge of the TSI distribution of the target population, hence assessing the representativeness of the validation sample can be challenging.

Given some prior knowledge of the epidemiological context, however, one could address this evaluation bias by using weighted resampling methods [23] to match the TSI distribution of the validation dataset to that of the target population where the assay will be employed. In Figures H-M in S1 Text we show that this technique can, in principle, ameliorate evaluation biases due to an unrepresentative TSI distribution. Nevertheless, the strength of this method depends on the properties of the target population and validation sample at hand, as well as the nature of the assay.

This study makes a series of simplifying assumptions. For example, we assume a rather simple function for the evolution of the biomarker (i.e., within-host viral genetic diversity), that is, *d*(*t*). Further sensitivity analyses should be carried out using other functions with similar behavior. We believe, however, that the main messages regarding evaluation biases are robust to other forms of *d*(*t*) as long as their behavior is similar to the one used herein. In fact, several biomarkers, related to both viral diversity and serologic assays, used to estimate HIV infection recency show a temporal behavior akin to the one modeled herein [11, 18, 20, 21], and other authors have used similar functional forms to model biomarker evolution [5].

Another limitation of the study is that our classification model has only one predictor (i.e., viral diversity). It stands to reason, and it is well stablished, that adding other predictors and combining multiple assays can lead to higher classification accuracy [4, 10, 21]. We expect the effect of the TSI distribution on assay performance to be systematically ameliorated as the assay increases its predictive power via the use of better (and more) biomarkers.

In addition, our classification was done in a binary way based on a diversity cutoff measure, d*. This diversity cutoff, or *classifier*, was determined via the objective function max(sensitivity + specificity). Giving equal importance to sensitivity and specificity has the clinically desirable property of maximizing the overall correct classification rate [28]; however, it does not incorporate any assessment of the context-specific risks and benefits of overestimating or underestimating the number of recent cases. Moreover, in Figures C and D in S1 Text we show that this binary classification approach can over- or under-estimate the fraction of recently infected individuals in the population depending on the shape of the TSI distribution. Certainly, given a specific context, a different objective function can be proposed to address specific priorities. An alternative approach, which avoids having to define a classifier, is to estimate the probability of being classified as a function of the biomarker level *d*_*i*_, namely *p*(*d*). The fitted logistic curve provides this function. This approach is useful for a *population-level* estimation of the fraction of individuals recently infected [5]. However, we find that the shape of the TSI distribution also affects the shape of $\hat{p}\left(d\right)$ (see Figure E in S1 Text for details). Hence, changing the approach from *binary* classification to classification *probability* does not eliminate the influence of the TSI distribution on the evaluation of recency assays: the shape of the TSI distribution affects both the value of the classifier (i.e., the individual-level or clinical classification) and the estimated probabilities of being classified as recent as a function of the biomarker level (i.e., the population-level estimation of recency).

In essence, this work shows how and why conclusions about the performance of an HIV recency assay can significantly depend on the TSI distribution. We consider that evaluation biases should be acknowledged and properly addressed when proposing a new HIV recency assay. Furthermore, to yield meaningful predictions in practice, one must not only provide an assay that performs well for a particular validation dataset, but more importantly consider whether or not the TSI distribution of this dataset is representative of the setting where the assay will be employed. Differences in demographic and epidemiological dynamics (which can lead to different TSI distributions) requires that assays be evaluated and tailored in a context-specific manner, not expecting it to perform effectively in all settings.

## Supporting Information

S1 Text The supplementary material contains additional information regarding: 1) the impact of biomarker variability on assay performance; 2) the assay’s performance at different TSI categories for the simulated distributions; 3) the effect of the TSI distribution on the diversity biomarker cutoff; 4) the comparison of the binary classification approach (as we do in the main text) with a probabilistic approach to classifying individuals into recent or chronic; 5) the uncertainty around the assay performance at different TSI categories for the empirical distributions; and 6) the use of weighted resampling as a way to overcome, in some cases, the evaluation biases shown in this work.
(PDF)
