# Supplementary material for: Assessing Biases in the Evaluation of Classification Assays for HIV Infection Recency
Source: PLoS One. 2015 Oct 5;10(10):e0139735. doi: 10.1371/journal.pone.0139735 (PMC4593552; doi:10.1371/journal.pone.0139735)
Supplement: S1 Text — The supplementary material contains additional information regarding: 1) the impact of biomarker variability on assay performance; 2) the assay’s performance at different TSI categories for the simulated distributions; 3) the effect of the TSI distribution on the diversity biomarker cutoff; 4) the comparison of the binary classification approach (as we do in the main text) with a probabilistic approach to classifying individuals into recent or chronic; 5) the uncertainty around the assay performance at different TSI categories for the empirical distributions; and 6) the use of weighted resampling as a way to overcome, in some cases, the evaluation biases shown in this work. (PDF) [file pone.0139735.s001.pdf]

# Supplementary Material: Assessing biases in the evaluation of classification assays for HIV infection recency

Oscar Patterson-Lomba[1], Julia Wu[2], Marcello Pagano[1]

[1] Department of Biostatistics, Harvard School of Public Health
[2] Department of Epidemiology, Harvard School of Public Health

## The impact of TSI distribution *and* variability on algorithm performance

Increasing the standard deviation, $u$, of the error of the statistical model of within-host diversity, $d_i = f(t_i) + \epsilon_i(u)$, also increases the variability in the simulated data. Here we investigated the impact of this variability, in combination with different TSI distributions, on classification performance. Results are reported in Tables A and B. Comparing the AUC results *between* Tables A and B we can see that, as expected, when the variability in the data is higher, the algorithm performs systematically worse than with a data set with less variability. Note: in these tables PPV refers to positive predictive value, which is equivalent to precision.

| *Parametrization* | *AUC* | *Sensitivity* | *Specificity* | *Precision* |
|---|---|---|---|---|
| $(a, b)$=(3.5, 5.0) | 95 (93.1, 96.7) | 91.9 (83.8, 98.0) | 85.6 (77.7, 92.5) | 59.2 (47.5, 72.7) |
| $(a, b)$=(5.0, 3.5) | 98.2 (95.7, 99.6) | 99.1 (90.0, 100) | 93.6 (85.5, 98.8) | 29.8 (10.1, 60.4) |
| $(a, b)$=(1.5, 1.5) | 98.0 (96.9, 98.8) | 95.9 (90.8, 100) | 91.0 (85.5, 95.7) | 73.3 (61.5, 85.0) |
| $(a, b)$=(0.4, 0.4) | 99.4 (99, 99.8) | 97.5 (94.4, 100) | 95.8 (92.0, 98.7) | 92.9 (87.1, 97.8) |

**Table A.** Classification performance $u = 0.05$, sample size=500, recency at 6 months. The 95% prediction bounds are obtained from 1000 simulations.

| *Parametrization* | *AUC* | *Sensitivity* | *Specificity* | *Precision* |
|---|---|---|---|---|
| $(a, b)$=(3.5, 5.0) | 69.2 (63.3, 74.5) | 72.1 (50.0, 89.8) | 60.0 (38.8, 80.0) | 29.1 (22.3, 38.1) |
| $(a, b)$=(5.0, 3.5) | 76.3 (62.3, 88.5) | 82.0 (50.0, 100) | 68.9 (40.8, 91.1) | 6.3 (2.3, 13.0) |
| $(a, b)$=(1.5, 1.5) | 77.9 (72.9, 82.6) | 78.5 (62.4, 91.8) | 66.9 (53.3, 81.0) | 38.2 (30.1, 48.6) |
| $(a, b)$=(0.4, 0.4) | 86.8 (83.5, 89.8) | 85.1 (75.9, 93.9) | 75.4 (65.3, 85.1) | 66.4 (58.5, 75.4) |

**Table B.** Classification performance $u = 0.2$, sample size=500, recency at 6 months. The 95% prediction bounds are obtained from 1000 simulations.

## Algorithm accuracy as a function of TSI: results for synthetic datasets

The results comparing mean performance using the four synthetic TSI distributions are depicted in Figure A, when the sample size is 1000 subjects, and the category period is 2 months (i.e., $\tau_{k+1} - \tau_k = 60$ days, $\forall k$).

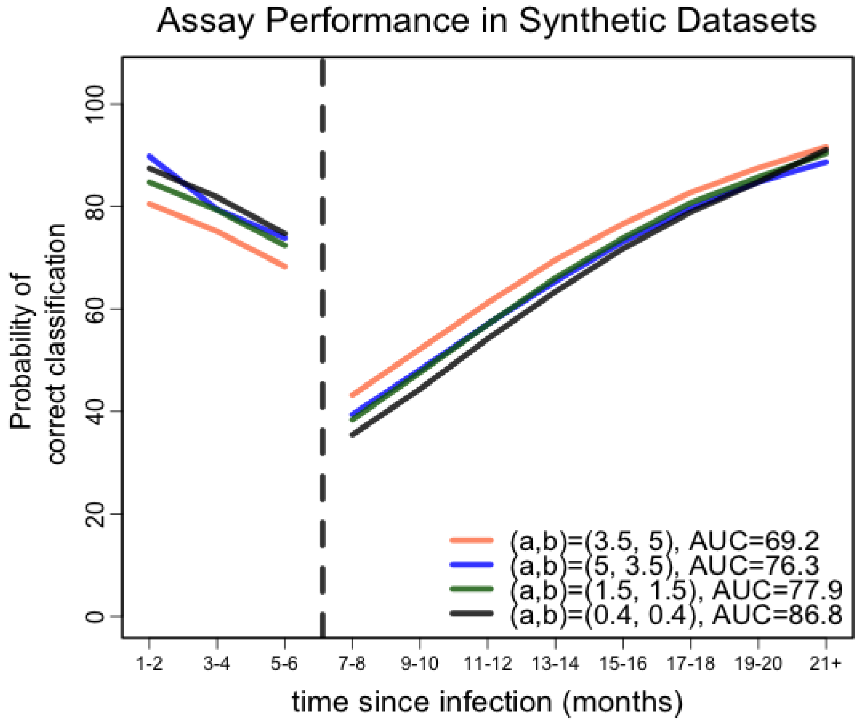


**Figure A.** Comparing the mean sensitivity (values before vertical dashed line) and the mean specificity (values after vertical dashed line) with recency at 6 months, sample size=1000, $u = 0.2$.

We can see that the *mean* probability of correctly determining recency status is not greatly affected by the shape of the synthetic TSI distributions (at least for the four distributions we considered). Notice, however, the difference between the orange (right-skewed, and worst AUC) and the black (bimodal, and best AUC) risk curves, whose underlying TSI distributions are quite similar to the empirical ones. We can clearly see that there is a performance trade-off between these two scenarios, with the bimodal curve performing better in terms of sensitivity but worse, in comparison, in terms of specificity.

# Effect of TSI distribution on the value of the diversity cutoff $d^*$

To show how the estimation of $p(t)$ (the probability of correctly classifying an individual with time-since-infection $t$) depend on the TSI distribution, lets formally express it:

$$p(t) = P[d < d^*|t] \quad \text{for } t < t^* \text{ and } p(t) = P[d > d^*|t] \quad \text{for } t \geq t^*$$

where $d^*$ is the biomarker cutoff and $t^*$ is an arbitrary recency cutoff, e.g., 180 days. The TSI distribution affects the estimation of $p(t)$ because it plays a key role in determining the biomarker cutoff $d^*$. We show this in Figure B, where, for example, the average diversity

score cut-off was 0.27 using the D228 dataset versus 0.39 when using D561 (right panel).

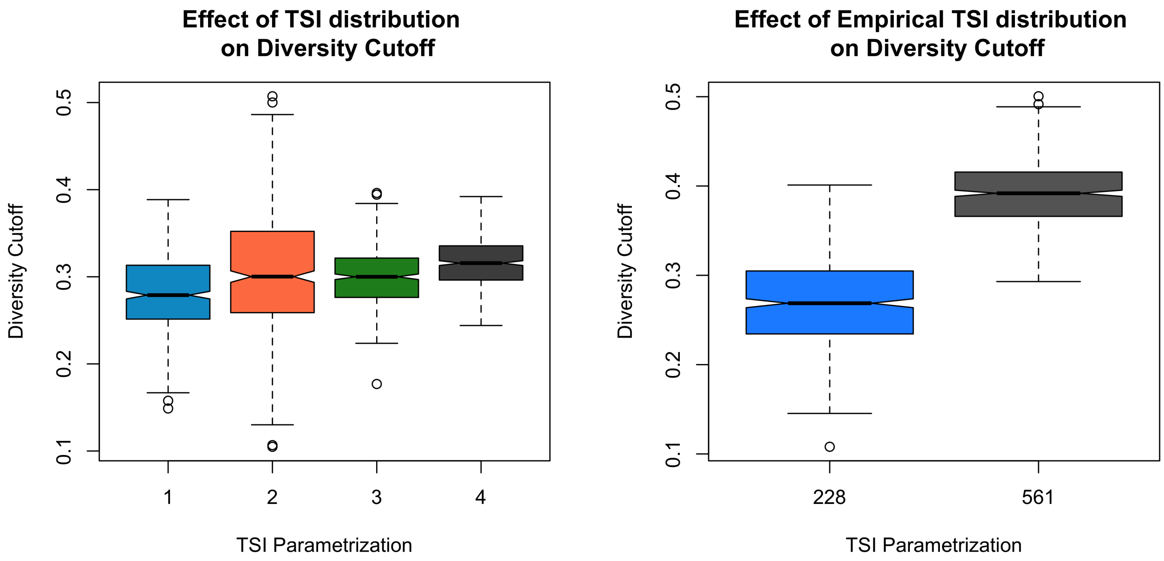


**Figure B.** Effect of the TSI distribution on the diversity biomarker cutoff ($d^*$). In the left panel we have used the four canonical TSI distributions (as in the main text) and in the right panel the two empirical TSI distributions D228 and D561. The figures show for each shape of the TSI distribution, the boxplots of 500 simulations using error u=0.2, and $t^*$=180 days. As the plots show, the TSI distribution can have a substantial effect on the selection of $d^*$, particularly when comparing the results using considerably different TSI distributions (as in the case of D228 versus D561), and therefore affect the shape of the estimated curve of $p(t)$.

## "Binary classification" versus "probability of being recent".

In the main manuscript we take the approach of classifying individuals in a binary way as recent or not based on comparison to a diversity cutoff measure, $d^*$. This diversity cutoff, or *classifier*, is determined via a cost/benefit analysis in which the objective function to be maximized is the sum of sensitivity and specificity

$$\max_d[\text{sensitivity}(d) + \text{specificity}(d)] \implies d^*.$$

Giving equal importance to sensitivity and specificity has the clinically desirable property of maximizing the overall correct diagnosis rate; however, it does not incorporate any assessment of the risks and benefits of overestimating or underestimating the number of recent cases. In other words, it does not take into account the epidemiological and socio-economic context in which the test would be applied. At the same time, that same lack of a specific

context limits our ability to propose a particular cost/benefit function, and thus we abstain from proposing one that gives more weight to sensitivity or specificity.

One way of avoiding having to define a *classifier* is by, instead of focusing on classifying an individual as recent or chronic, providing a probability of being classified as recent based on the biomarker level of such individual (or equivalently, its respective time since infection). The fitted logistic curve indeed provides for each biomarker value the respective *predicted* probability of being classified as recent. Granted, this approach is less desirable if our main objective is to correctly classify each individual. The latter approach is useful for a population level estimation of the fraction of individuals recently infected.

In this section we study how these two approaches differ from each other, and how focusing on binary classification (recent vs chronic) deviates from estimating the correct fraction of infected as a function of the classifier selected.

Let

$$f = \sum_i s_i/N$$

be the fraction of recent cases in the study population, where $s_i$ is the classification of individual $i$ as 1 if recent, or 0 if chronic (e.g., 1 if infected less than 180 days ago, else 0).

Also, let

$$\hat{f}_{bc} = \sum_i c_i/N$$

be the estimated or predicted fraction of recent cases as determined by the binary classification approach, where $c_i$ is the classification of individual $i$ as 1 (if classified as recent) or 0 (if classified as chronic). That is, for any classifier $d_c$, $c_i = 1$ if $d_i < d_c$, else, $c_i = 0$.

Finally, let

$$\hat{f}_p = \sum_i \rho_i/N$$

be the estimated or predicted fraction of recent cases as determined by the probabilistic approach, where $\rho_i$ is the probability of classifying an individual as recent. These probabilities are directly obtained from the fitted logistic function $\log[\rho_i/(1-\rho_i)] = \hat{\beta}_0 + \hat{\beta}_1 d_i$, with $\hat{\beta}_0$ and $\hat{\beta}_1$ estimated from regressing status $s$ on diversity $d$.

Figures C and D compare the two aforementioned approaches in estimating the fraction of the study population that is recently infected, with recency defined at 180 days and 365 days, respectively. The predicted fraction of recent cases estimated by the binary classification, $\hat{f}_{bc}(d_c)$, increases with the diversity cut-off or classifier. Conversely, $\hat{f}_p$ does not depend on $d_c$, and as expected it is in fact equal to the actual fraction of recent cases $f$.

In green is the value of $\hat{f}_{bc}$ at $d^*$ (i.e., the classifier that maximizes sensitivity + specificity). When recency is defined at 180 days (Figure C), $\hat{f}_{bc}(d^*)$ overestimates $f$. In contrast, when recency is defined at 365 days (Figure D), $\hat{f}_{bc}(d^*)$ overestimates $f$ in the case of declining epidemics, but underestimates it in the case of emerging epidemics. In general, we find that $\hat{f}_{bc}(d^*) > f$ if $t^* < mean(tsi)$, whereas $\hat{f}_{bc}(d^*) < f$ if $t^* > mean(tsi)$, where $t^*$ is the time point dividing recent from chronic cases and $tsi$ is the times since infection of all individuals in the sample (i.e., mean of the TSI distribution). Moreover, if the objective function is changed to

$$\max_d [w_1 \times \text{sensitivity}(d) + w_2 \times \text{specificity}(d)] \implies d^*,$$

where $w_1 = f$ and $w_2 = 1 - w_1$, then we get that $\hat{f}_{bc} \to f$. This last approach implies that, instead of giving equal weights to sensitivity and specificity (i.e., $w_1 = w_2$), sensitivity (specificity) is given a weight corresponding to the fraction of people in the sample that are recent (chronic).

Notice also that the difference $|\hat{f}_{bc}(d^*) - f|$ is smaller for the case of bimodal epidemics. It is also worth noting that the fraction defined as $\hat{f}_{ps} = \sum_i (\rho_i | s_i = 1)/N$ is always less than $\hat{f}_p$ and $f$. That is, the estimated recent fraction is underestimated if we only account for the recency probability of individuals that are actually recently infected.

Hence, whether the binary classification approach over or underestimates the fraction of recently infected individuals in the population depends on how we define recency and the epidemiological context (shape of the TSI distribution).

One could argue that, to estimate the fraction of recently infected individuals in a study population, instead of providing a classifier $d^*$– which is sensitive to the objective function used– one could provide the coefficients $\hat{\beta}_0$ and $\hat{\beta}_1$ of the log odds function $\log[\rho_i/(1-\rho_i)] = \hat{\beta}_0 + \hat{\beta}_1 d_i$. With this information, given a diversity (i.e., biomarker) level one can estimate the $p_i$'s of the individuals and from those estimate the recently infected fraction. However, the shape of the TSI distribution also affects the estimation of $\hat{\beta}_0$ and $\hat{\beta}_1$. Figure E shows how the function $\rho(d)$ differs between the four epidemic scenarios explored before.

We also estimate the probability of correct classification at time $\tau$, namely $\phi(\tau)$, using the probabilistic approach, in contrast to $p(\tau)$ in the main text which was estimated through the binary classification approach. In fact, as presented in the main text, we estimate the discrete version of $\phi(\tau)$, namely $\hat{\phi}_k$, for individuals that belong to the $k^{th}$ TSI category. To do so we again use the predicted probabilities $\rho_i$ obtained from the logistic fit of *status* $\sim$ *diversity*. Thus, the estimate of $\hat{\phi}_k$ via the probabilistic method is given by

$$\hat{\phi}_k = \frac{\sum_{i \in C_k} \rho_i}{n_k} \tag{1}$$

where $C_k$ is the set of individuals with $t_i \in (\tau_k, \tau_{k+1})$ and $n_k$ is the total number of people in such set. Note again that we do not need to specify a classifier $d^*$ to estimate $\hat{\phi}_k$. As for the estimates of $\hat{p}_k$, we can also explore how does $\hat{\phi}_k$ depend on the TSI distribution.

Figure F shows that the TSI distribution affects both the probability of correctly classified as recent (or chronic) (i.e., $\hat{p}_k$, as determined by the binary classification approach) and the probability of being correctly classified (i.e., $\hat{\phi}_k$, as determined by the probabilistic approach), given time since infection. Therefore, the TSI distribution affects assay performance regardless of how we measure this performance.

## Variability around the estimates of $\hat{p}_k$

The variability around the estimates of $p_k$ in a given TSI group of bin $k$ depend on the number of observations in that bin. Moreover, if the objective is to maximize the overall accuracy of the assay, then those bins that contain a large fraction of the observations will

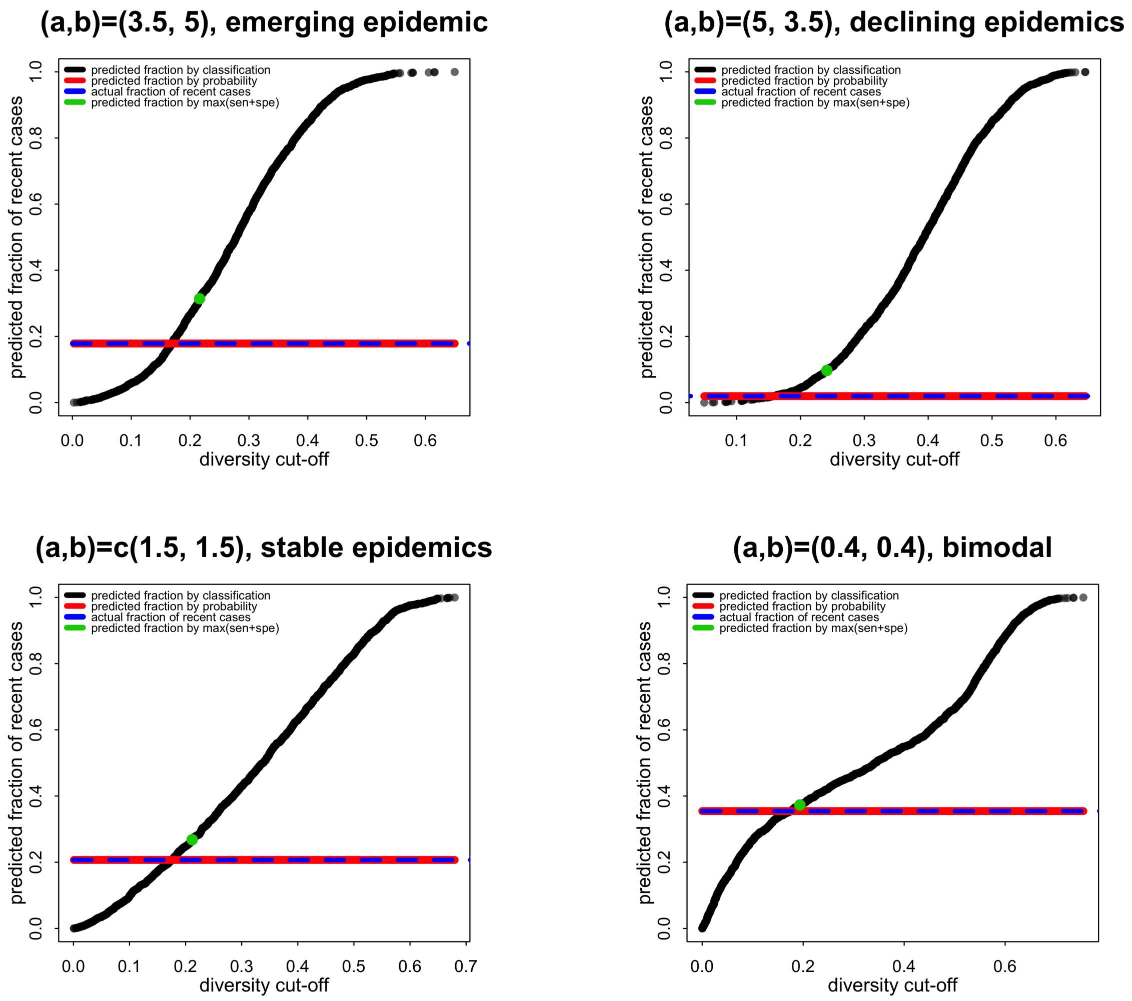


**Figure C.** Comparing the two approaches of classification: $\hat{f}_{bc}$ in black, $\hat{f}_p$ in red and $f$ in dashed blue. In green is the value of $\hat{f}_{bc}$ at $d^*$ (i.e., the classifier that maximizes sensitivity + specificity). Recency defined at 180 days, TSI range of $\Delta T = 700$ days, sample size =2000.

get more unbiased estimates of $p_k$. The reason being that how one defines the biomarker cutoff values depends greatly on the shape of the TSI distribution, not just the amount of data in each bin. In other words, the variability around the estimates of $p_k$ in a given bin will depend more on the *absolute* number of observations in that bin, whereas the amount of bias of the $p_k$ estimates in a given bin will depend more on the number of observations in that bin *relative* to other bins. Given that the true value of $p_k$ is 1 for all $k$, the amount of bias in accuracy in each bin can be quantified by $1 - \hat{p}_k$.

Using the binary classification approach, as in the main text, Figure G illustrates how the bias and the variability of $\hat{p}_k$ tends to be lower for highly represented bins, and vice versa.

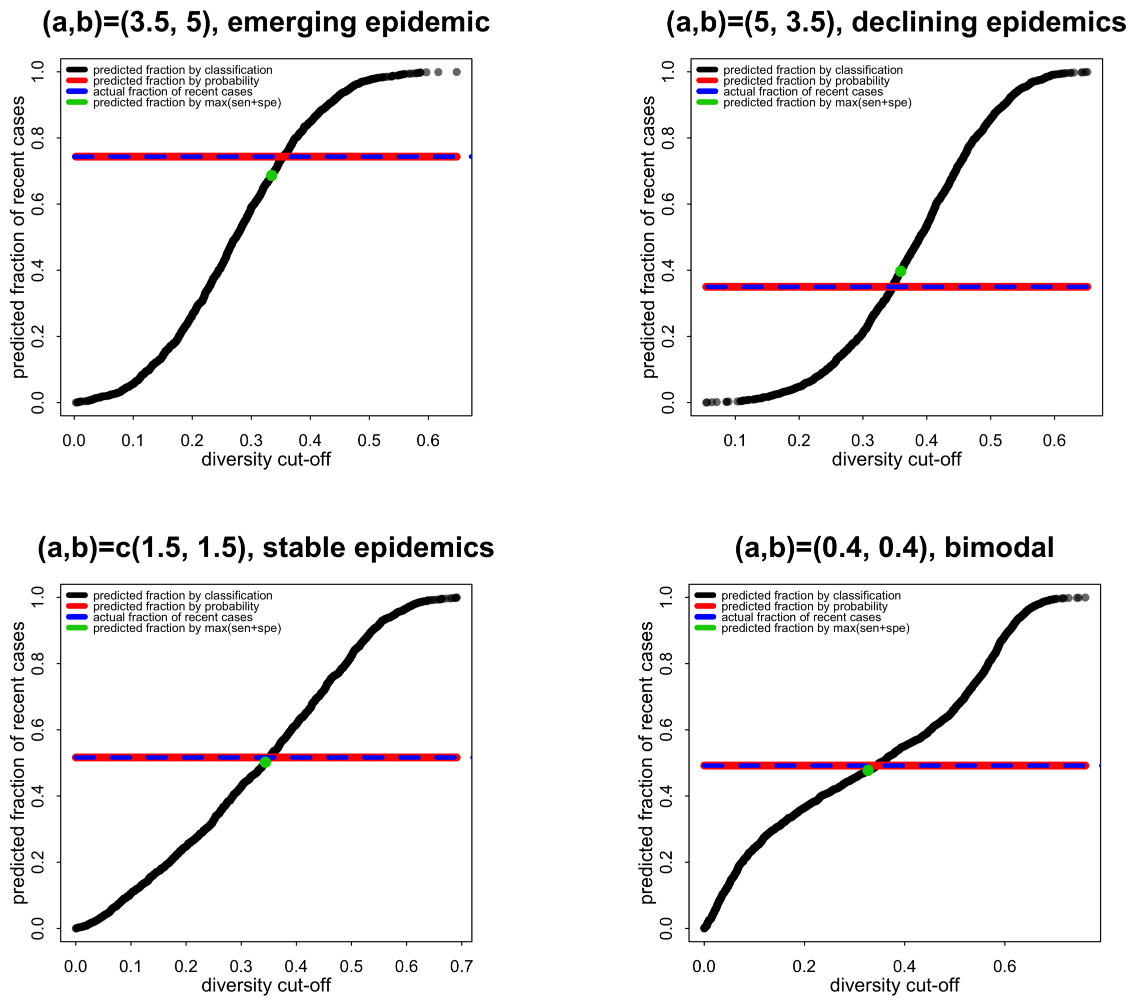


**Figure D.** Comparing the two approaches of classification: $\hat{f}_{bc}$ in black, $\hat{f}_p$ in red and $f$ in dashed blue. In green is the value of $\hat{f}_{bc}$ at $d^*$ (i.e., the classifier that maximizes sensitivity + specificity). Recency defined at 365 days, TSI range of $\Delta T = 700$ days, sample size =2000.

# Resampling to Match Sampled and Target Distributions

We have shown that the shape of the TSI distribution of the validation data can have significant effects on the evaluation of recency estimation assays. At the same time, the TSI distribution may not be representative of the *target* population in which the assay will be applied. As a result, if these distributions differ then the evaluation of the assay using the validation sample may be biased [1]. Here we propose a way to counteract these effects, given a prior knowledge of the epidemiological context.

Let the *sampled* TSI distribution be the TSI distribution of the validation dataset (also known as *study population*). Also, let the *target* TSI distribution be the TSI distribution in a given context (e.g., region, country) of interest. Suppose the shape of the *target* TSI

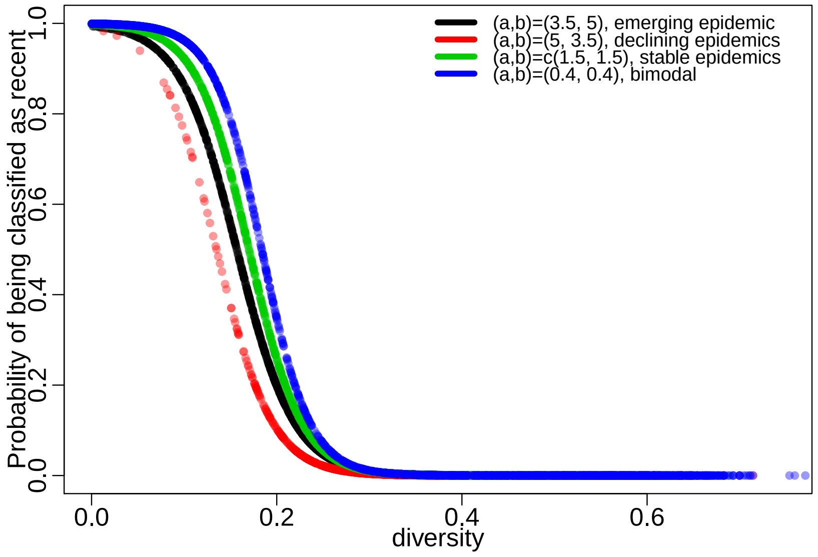


**Figure E.** Probability of being classified as recent as a function of diversity, for the four different epidemic scenarios. Recency defined at 180 days, TSI range of $\Delta T = 700$ days, sample size =2000.

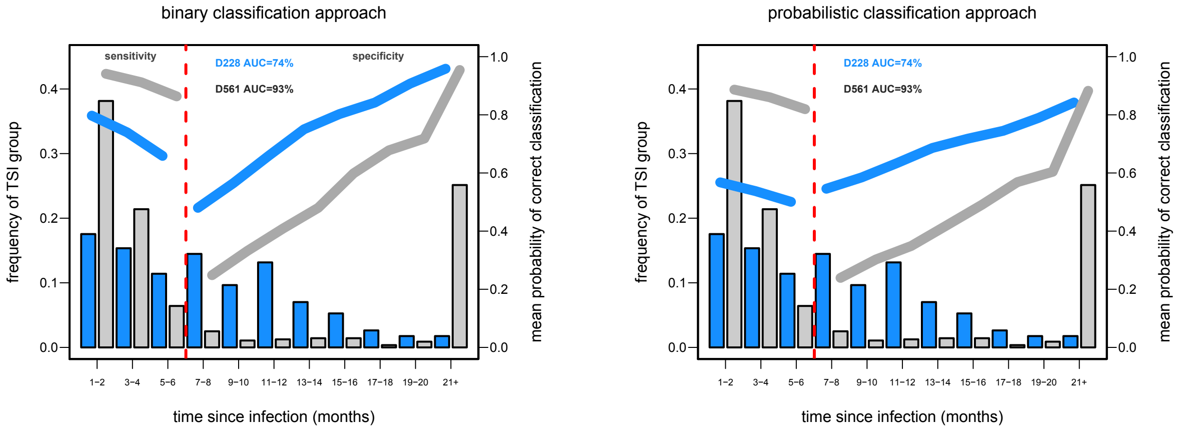


**Figure F.** Comparing the mean performance of the same hypothetical HIV recency assay using the two empirical TSI distributions and using the binary classification approach (left panel) versus the probability classification approach (right panel). Note the different $y$ axis, with the left $y$ axis representing the frequency of each of the 2-month TSI groups, and the right $y$ axis representing the mean performance (as in the main text). We see that the mean performance is similar in both panels using the two different approaches. Parameter values: $u = 0.2$, $h = 500$.

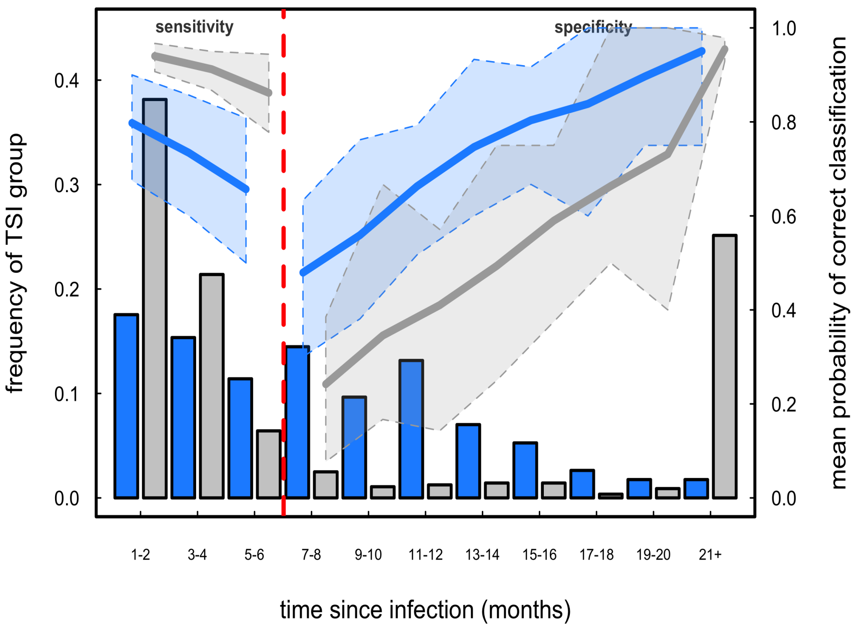


**Figure G.** Prediction bounds (75%) around the estimates of $p(t)$ for 500 simulations. The parameter values are the same as those used to generate Figure 8 in the main text where we employed the TSI distributions from the empirical datasets D228 (blue) and D561 (gray). The bias and the variability of $p_k$ tend to be lower for highly represented bins.

distribution is roughly known (which might often not be the case since our estimation exercise is precisely aimed at quantifying these epidemiological scenarios), then one way to offset these evaluation biases is by using weighting resampling from the *sampled* TSI distribution at hand to match the *target* distribution.

To this end, and like in the main manuscript, we explore different epidemic scenarios that can be found in reality in combination with possible *sampled* TSI distributions structures. Once the resampling is done, the classification method can then be assessed for each combination of *target* and *sampled* TSI distributions. The basic premise behind this approach is that to make fair assessments of recency assays these should be compared and evaluated using datasets with similar TSI distributions, and when the epidemiological context is at least partly known, the TSI distributions of the validation datasets should resemble the ones from the context of interest.

## Weighted sampling method

The goal here is to, from the validation dataset at hand, construct a representative sample (in terms of its TSI distribution) of the epidemiological scenario of interest (i.e., the target population). The first matching is in terms of the TSI ranges or support of the two distributions, and we do so by constraining the ranges to the least extensive. Hence, the range of

the new sampled distribution would be

$$[\max(\min(target\ TSI,\ \ sampled\ TSI)), \min(\max(target\ TSI,\ \ sampled\ TSI))].$$

Once the ranges have been matched, in order to draw from the *sampled* population as to mimic the *target* population, we must compute the sampling weights of each of the strata as the inverse of the ratio between the *target* and the *sampled* distributions for each TSI strata. As in stratified sampling designs [1], we define the different strata or TSI groups, with strata being mutually exclusive (non-over lapping). For example, equally spaced time intervals (e.g., 0-1 month, 1-2 months...). Thus, if the population, of size $N$, consists of $k$ discrete elements, then under stratified sampling, $N = \sum_{i=1}^{k} N_i$.

Hence, after assigning individuals to $k$ different TSI strata of categories both in the *target* distribution and in the *sampled* distribution, the probability of sampling from the $k_{th}$ category is given by

$$s_k = \frac{(\text{frequency of individuals in } k_{th} \text{ category in the } \textit{target} \text{ population})/N_{target}}{(\text{frequency of individuals in } k_{th} \text{ category in the } \textit{sampled} \text{ population})/N_{sampled}}.$$

Note that since $S = \sum_k s_k$ is not necessarily equal to 1, the $s_k$'s are considered *relative* probabilities. To transform these into *absolute* probabilities we divide each $s_k$ by $S$.

Once the sampling probabilities of each TSI categories are computed, then we can begin constructing the *sampled* distribution. Once a given TSI category has been selected for sampling, we draw at random and with replacement from the samples in that category. We perform this last steps (select a category and then sample with replacement from it) a given number of times $r$. For instance, we select $r$ to be equal to the number of samples in the *sampled* distribution. After repeating this procedure $r$ times, we have a *resampled* distribution that should match the *target* one.

As we see in the figures below, this resampling procedure yields *resampled* distributions that in many cases resemble that of the *target* one. In some cases, however, the resulting distribution is not a good representation of the *target* one. This occurs especially when the values of $s_k$ are relatively large (i.e., frequency in the sampled population is much lower than in the target one) and as result one category gets selected too often, rendering a mismatched *resampled* distribution. Note how this is generally the case when the *sampled* distribution is skewed either to the left or right (orange and blue distributions in the figures).

In the figures below, we have assumed that the TSI ranges are 600 days (sample size=20,000) and 1000 days (sample size=10,000) for the *target* and *sampled* distributions, respectively, except for the case of the empirical distributions.

We use the empirical data to show how this resampling procedure can reduce the evaluation bias due to mismatching of the TSI distributions of the *target* and *sampled* populations. Specifically, we use D561 as the *sampled* population and D228 as the *target* population (as in Figure L), and the diversity evolution model $h(t)$ described in the main manuscript. In short, for each simulation run, we determine the diversity measures for each TSI in the D561 sample, and compute the respective performance metrics (AUC, sensitivity, specificity and PPV). We then resample from the TSI distribution of D561 as to match that of D228, and again compute the performance metrics using the *resampled* population. To compare

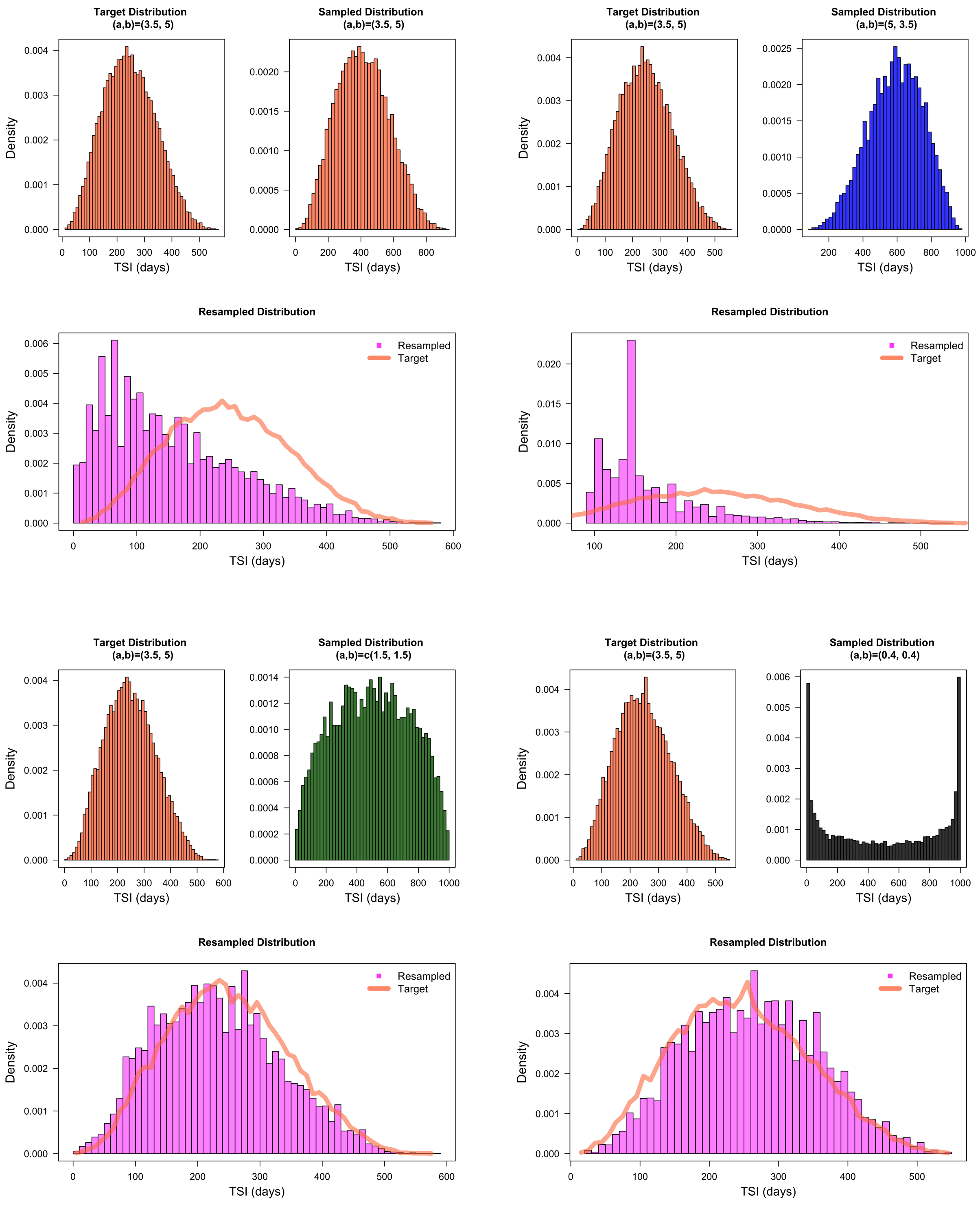


**Figure H.** Resampling from different *sampled* distributions to match a “rising” epidemic scenario.

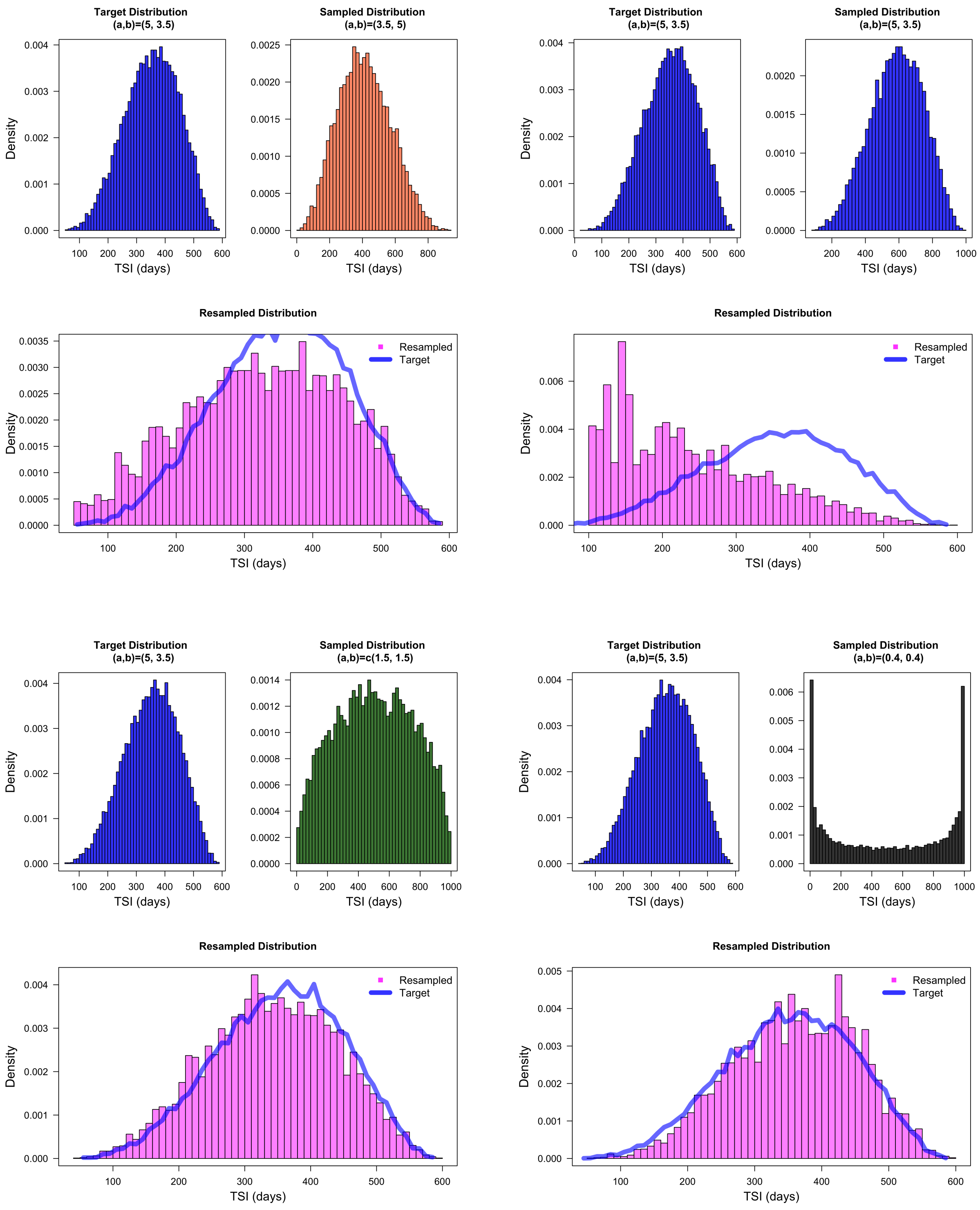


**Figure I.** Resampling from different *sampled* distributions to match a "declining" epidemic scenario.

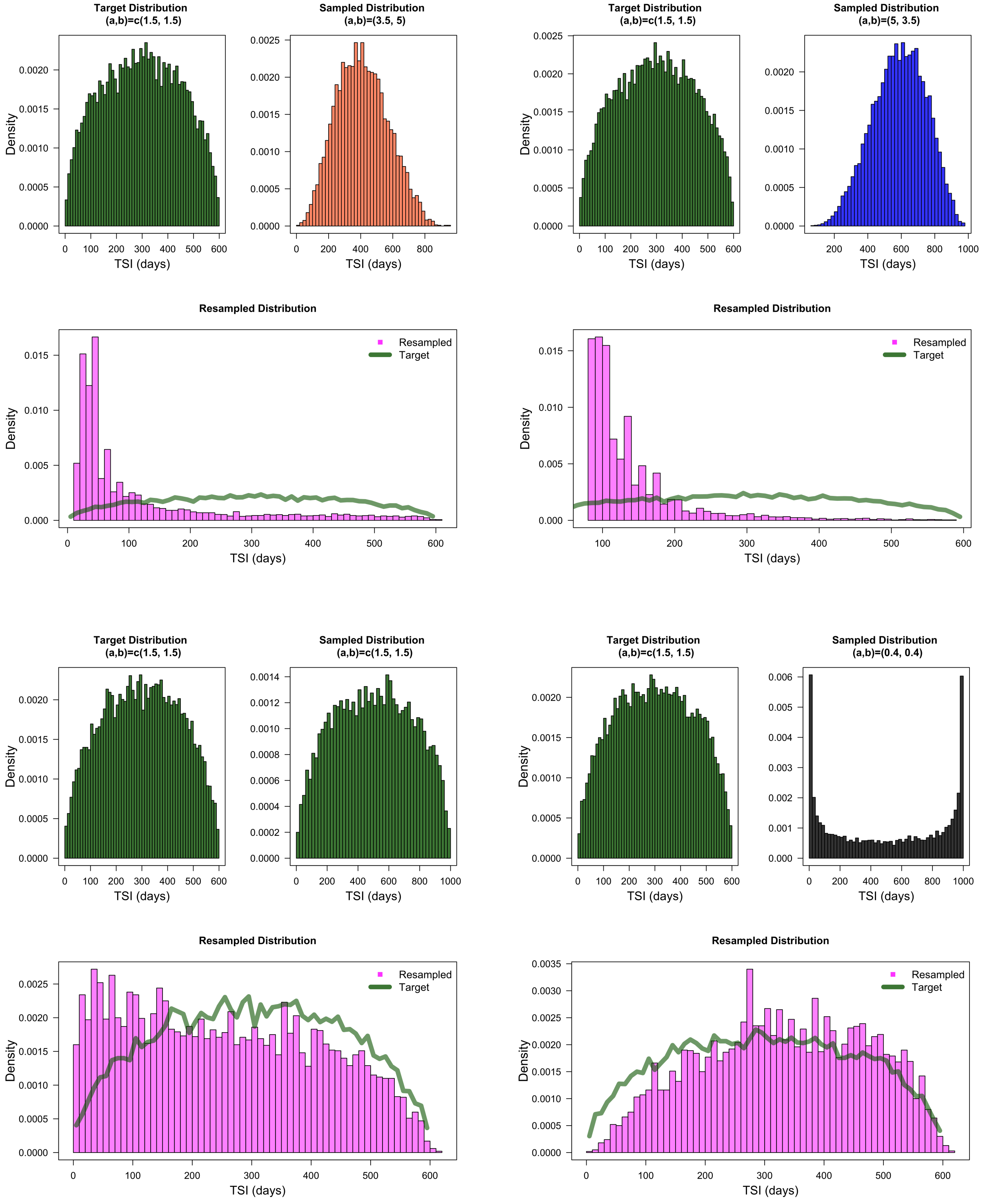


**Figure J.** Resampling from different *sampled* distributions to match a "stable" epidemic scenario.

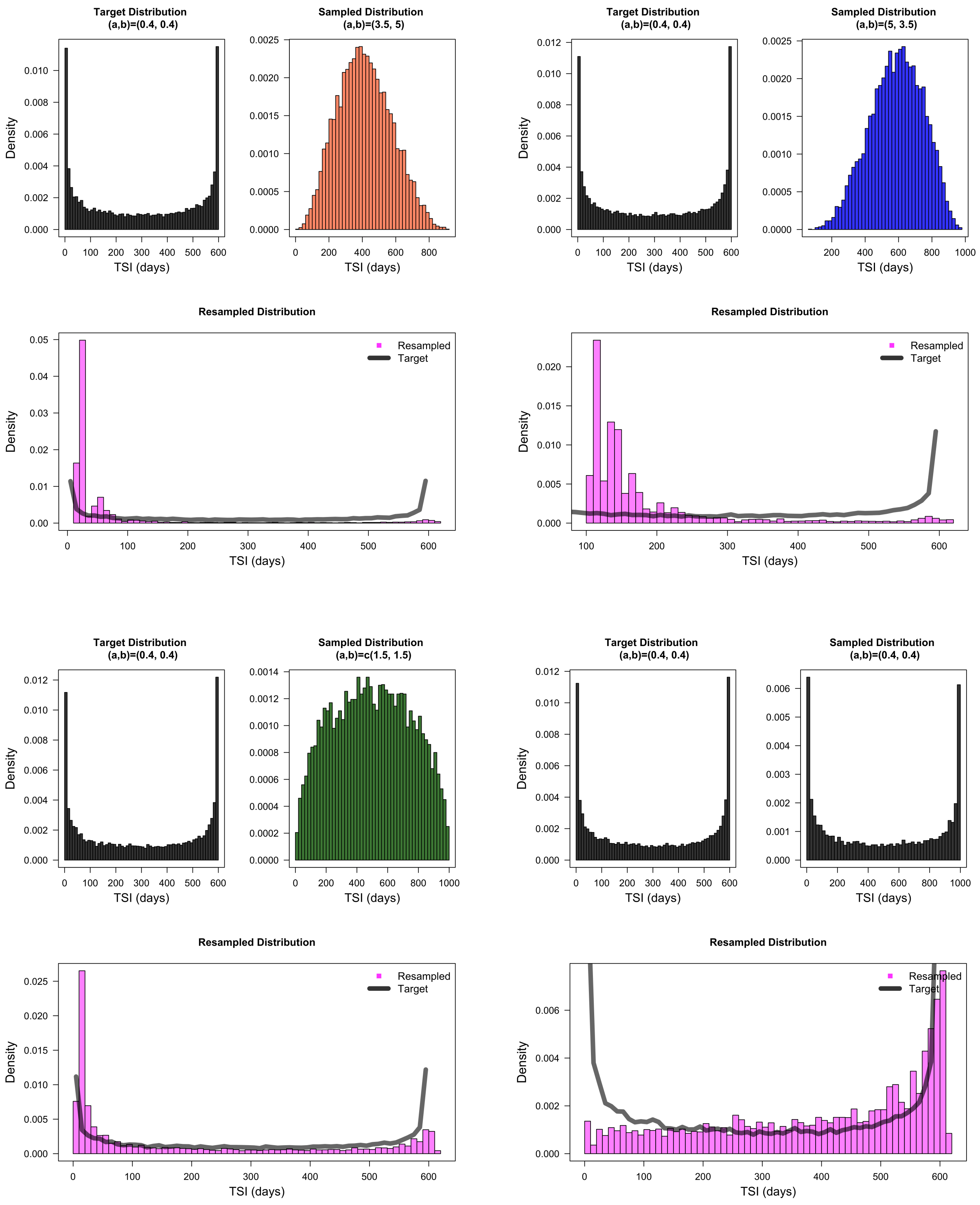


**Figure K.** Resampling from different *sampled* distributions to match a "bimodal" epidemic scenario.

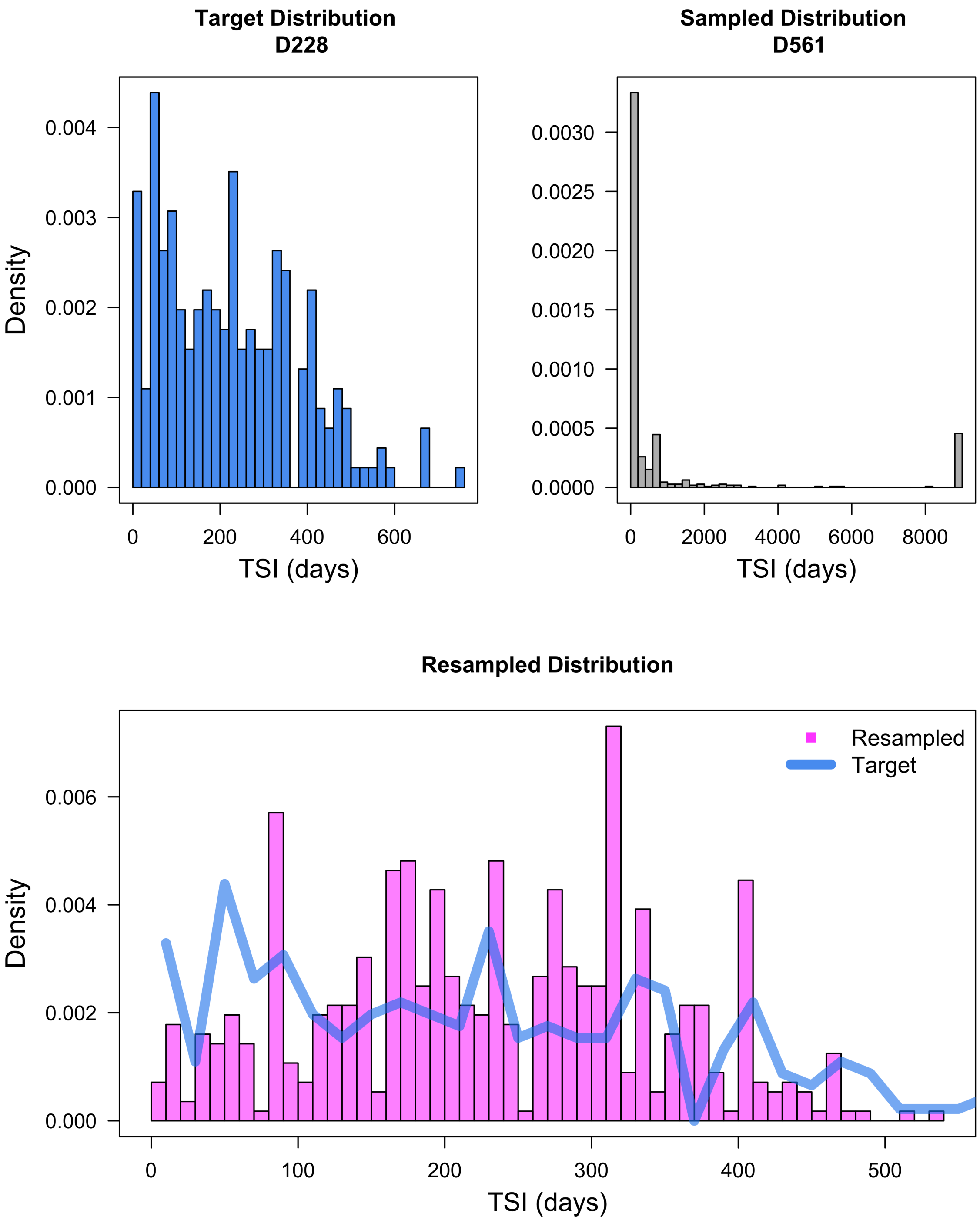


**Figure L.** Resampling from the D561 distribution to match the D228.

these performance metrics with the ones obtained assuming that the TSI distribution comes from the target population, we compute the performance metrics using the D228 dataset. We repeat this procedure 1000 times to get means and prediction intervals for each of the metrics.

As we can see from Figure M, the performance metrics obtained using the *resampled* population are quite similar, in terms of means and prediction intervals, to those obtained using the *target* population as a validation dataset. This indicates that indeed, this resampling method can, in principle, reduce evaluation biases when the TSI distribution of the *sampled* population is not representative of the *target* population.

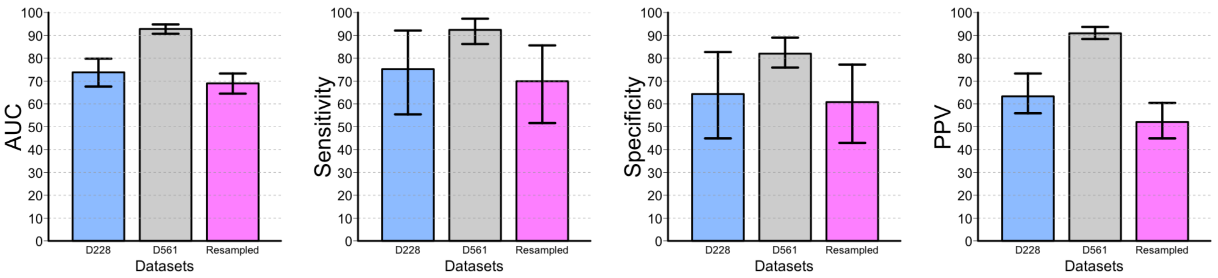


**Figure M.** Reducing evaluation bias through resampling. Classification performance $u = 0.2$, sample size=500, recency at 6 months. The 95% prediction bounds are obtained from 1000 simulations.

There are, however, a number of issues with this approach. Maybe the most concerning one is that since resampling is done with replacement, samples that belong to a category with relatively high $s_k$ will be sampled several times, possibly introducing spurious correlations among the samples in the *resampled* distribution due to repeated measurements. How problematic this issue is depends largely on the nature of the assay (e.g., is the assay sensitive to repeated measurements?). One way to avoid this issue is by resampling without replacement, but that limits our ability to match the *target* distribution. Another aspect to consider is the stratification (or binning) of the distributions, as different binning structures could in principle lead to less efficient matching outcomes.

Clearly, a limiting aspect of this approach is the amount of information at hand, both in the *target* and *sampled* TSI distributions. The more information (more data) we have on these distributions, the more robust the sampling probabilities will be and the less problematic the sampling with replacement issue will be, hence, the more stable the resampling method will be.

All in all, this method does not guarantee completely overcoming the issue of evaluation biases due to mismatching TSI distributions. The strength of this method will depend on the properties of the target and study population at hand. This method represents, however, a valid strategy to ameliorate some of the potential evaluation biases in recency estimation. It is beyond the scope of this paper to provide specific recommendations for how to solve this difficult and contextual problem.

# References

1. Pagano M, Gauvreau K (2000) Principles of biostatistics, volume 2. Duxbury Pacific Grove, CA.
